# Supplementary material for: The Potential of a Saliva Test for Screening of Alveolar Bone Resorption
Source: Healthcare (Basel). 2023 Jun 21;11(13):1822. doi: 10.3390/healthcare11131822 (PMC10340156; doi:10.3390/healthcare11131822)
Supplement: Supplementary file 1 [file healthcare-11-01822-s001.zip › healthcare-2428001-supplementary.pdf]

Table S1. Primer sequence.

Bacteria	Sequence (5' – 3')
P. gingivalis	F: AGG CAG CTT GCC ATA CTG CG
	R: ACT GTT AGC AAC TAC CGA TGT
T. denticola	F: TAA TAC CGA ATG TGC TCA TTT ACA T
	R: TCA AAG AAG CAT TCC CTC TTC TTC TTA
T. forsythia	F: GCG TAT GTA ACC TGC CCG CA
	R: TGC TTC AGT GTC AGT TAT ACC T

Table S2 Accuracy of screening at the cutoff point of the bone crest level at 3mm

BCL, bone crest level; LR+, a positive likelihood ratio; LR-, a negative likelihood ratio. The values were obtained using the explanatory variables of the risk factor of periodontitis, except for the number of teeth.

BCL	3.0 mm	
	Positive	Negative
True	111	667
False	167	32
Sensitivity	0.78	
Specificity	0.80	
LR+	3.88	
LR-	0.28	

Table S3 Likelihood ratio test at bone crest level at 3.0 mm, except number of teeth from the explanatory variables

The explanatory variables were categorized into multiple groups. The number of residual teeth was excluded from the explanatory variables. (*) indicates the statistically significant difference with a *p-value* of <0.05.

LR, a likelihood ratio; *P.g*, *Porphyromonas gingivalis*; *T.d*, *Treponema denticola*; *T.f*, *Tannerella forsythia*

Explanatory variables	3.0 mm	
	χ^2 for LR	*p-value*
Age	93.95	<.001*
Gender	1.32	0.251
Smoking habit	15.12	<.001*
Stimulated saliva volume	21.32	<.001*
log (Bacterial count of *P.g* + 1)	23.53	<.001*
log (Bacterial count of *T.d* + 1)	5.77	0.056
log (Bacterial count of *T.f* + 1)	0.52	0.773

.

Table S4 Multiple logistic regression analysis at the cutoff point of the bone crest level at 3.0 mm, except the number of teeth from the explanatory variables.

Adjusted odds ratios (ORs), 95% confidence interval (CI), and p-value were obtained from the multiple logistic regression analysis of the risk factor of periodontitis, except for the number of teeth. Variables were categorized into multiple groups. (*) indicates the statistically significant difference with a p-value of <0.05.

P.g. Porphyromonas gingivalis; *T.d*, *Treponema denticola*; *T.f*, *Tannerella forsythia.*

Variable	ORs	95% CI Lower	95% CI Upper	*p-value*
**Age**				
less than 30	1.00 (reference)			
30-39	4.90	1.87	16.89	<.001*
40-49	12.13	4.57	42.20	<.001*
more than 50	33.77	12.78	117.43	<.001*
**Gender**				
Male	1.00 (reference)			
Female	0.77	0.49	1.21	0.251
**Stimulated saliva volume**				
More than 15 ml	1.00 (reference)			
5 - 15 ml	15.22	3.07	278.27	<.001*
Less than 5 ml	25.28	4.79	472.11	<.001*
**Smoking habit**				
Non-smoker	1.00 (reference)			
Current smoker	2.95	1.72	5.03	<.001*
**Log(Bacterial count of *P.g* + 1)**				
Less than 5	1.00 (reference)			
5 to 7	2.30	1.48	3.58	<.001*
More than 7	5.42	2.38	12.47	<.001*
**Log(Bacterial count of *T.d* + 1)**				
Less than 5	1.00 (reference)			
5 to 7	1.58	0.98	2.59	0.062
More than 7	2.68	1.08	6.28	0.034*
**Log(Bacterial count of *T.f* + 1)**				
Less than 5	1.00 (reference)			
5 to 7	0.91	0.58	1.44	0.711
More than 7	1.16	0.60	2.19	0.649

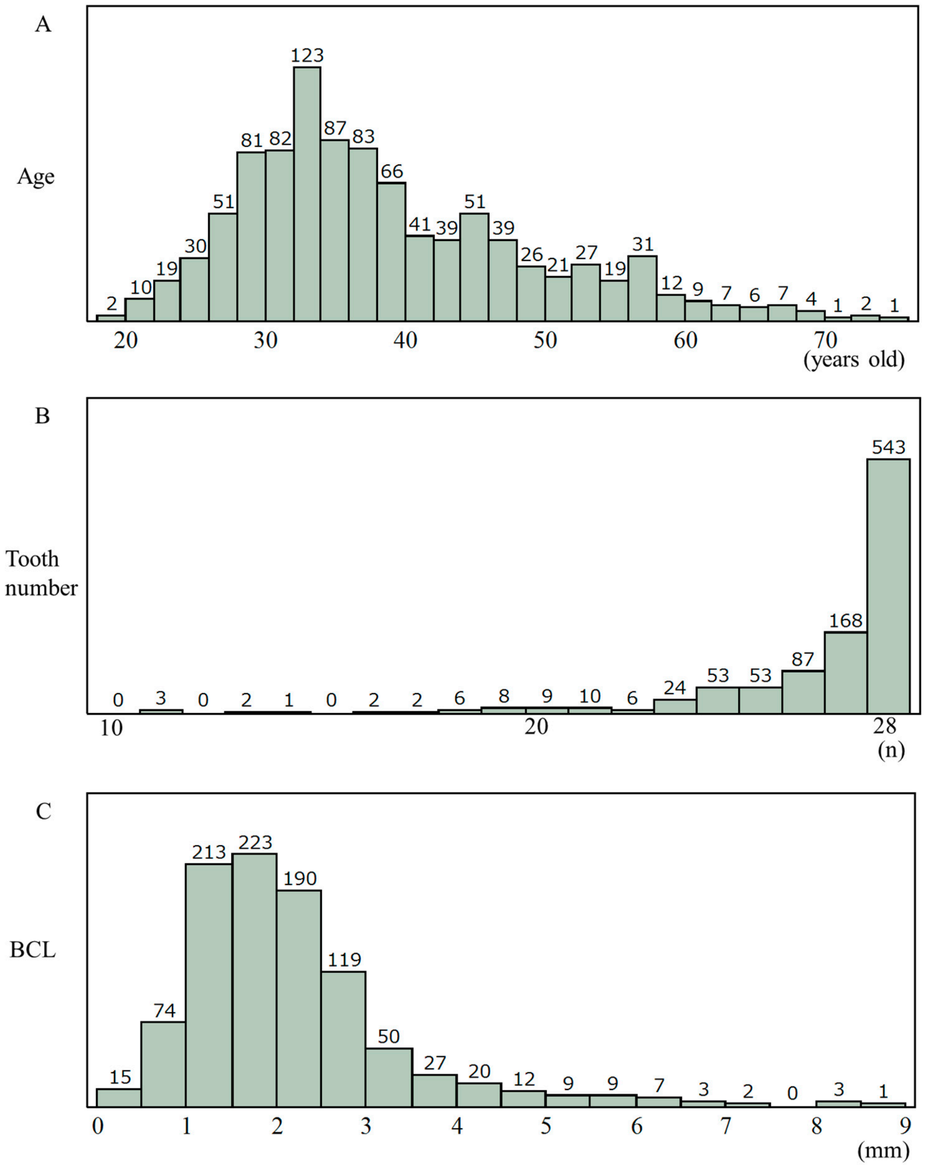

Figure S1 Distribution of patient age, number of residual teeth, and bone crest level. The histograms present approximate representations of the distribution of the characteristics, in terms of age (A), number of teeth (B), and bone crest level (C) with the following class intervals: 2 years old, one tooth, and 0.5 mm, respectively. BCL, bone crest level.

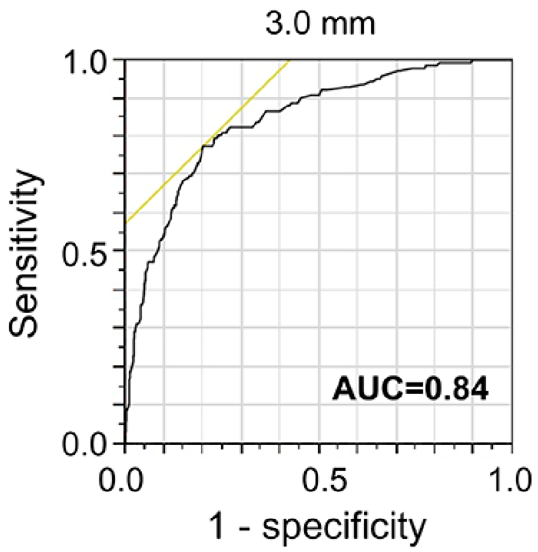

Figure S2 Receiver operating characteristic (ROC) curves at bone crest level at 3.0mm by multiple clinical parameters except for the number of teeth.

The ROC curves were plotted at the BCL of 3.0 mm using explanatory variables of the risk factor of periodontitis, except the number of teeth. The area under the ROC curve value is present at the bottom right of the figure.
